# Learning about the genetic risk in my family: Preferences of Italian nurses

**DOI:** 10.1007/s12687-026-00892-w

**Published:** 2026-05-11

**Authors:** Lea Godino, Linda Battistuzzi, Daniela Turchetti, Liliana Varesco, Vanessa Gentili, Paolo Chiari, Alvisa Palese

**Affiliations:** 1 Medical Genetics Unit, IRCCS Azienda Ospedaliero-Universitaria Di Bologna, Bologna, Italy; 2 Department of Biomedicine and Prevention, University of Rome “Tor Vergata”, Viale Montpellier, 1, 00128 Rome, Italy; 3 Medical Oncology Unit 2, IRCCS Ospedale Policlinico San Martino, Genoa, Italy; 4 Department of Medical and Surgical Sciences, University of Bologna, Bologna, Italy; 5 Unit of Hereditary Cancer, IRCCS Ospedale Policlinico San Martino, Genoa, Italy; 6 Department of Medicine, University of Udine, Udine, Italy

**Keywords:** Nurses, Genetic literacy, Genetic risk, Genetic information, Moral responsibility

## Abstract

This study explores Italian nurses’ preferences and views regarding the disclosure and sharing of genetic risk information within families, focusing on their genetic literacy and responsibility, and comparing these perspectives with laypeople. A nested cross-sectional online survey, originally designed for the general population, included three hypothetical scenarios of inherited genetic risk (Cystic Fibrosis, Hereditary Cancer Syndromes, and early-onset Alzheimer’s disease) and items assessing genetic literacy (awareness), family cohesion, disclosure preferences, and responsibility. Quantitative data were analyzed descriptively and inferentially, and qualitative responses were analyzed inductively using Reflexive Thematic Analysis. Among the 1,302 respondents, 501 were healthcare professionals (HCPs), including 315 nurses. Their genetic literacy was modest (mean 2.5/4), higher than laypeople’s, with no differences by age or education. Nearly all (94.9%) wished to be informed of genetic risk for at least one condition, with 75.9% preferring to be informed for all three, similar to laypeople (78.1%). Most (97.8%) would undergo genetic testing if informed (95.6% of laypeople). Responsibility for disclosure was viewed as shared between relatives and HCPs (39.1%) or by everyone involved (themselves, relatives and HCPs) (25.1%), echoing laypeople’s views. Qualitative findings from the few who did not wish to be informed (*n* = 16) emphasized anxiety, psychological self-protection, and scepticism toward predictive medicine, similar to laypeople. Italian nurses expressed views and preferences similar to laypeople. While they valued receiving genetic risk information and HCP involvement in family communication, limited familiarity with clinical applications of genetic testing highlights a gap in nursing education and practice, with implications for targeted training.

**Supplementary Information:**

The online version contains supplementary material available at 10.1007/s12687-026-00892-w.

## Introduction

Genetic testing allows the detection of inherited disorders and, for certain conditions, enables targeted prevention strategies. Identification of a pathogenic variant in a proband also facilitates cascade genetic testing, whereby testing is offered to biological relatives, so they, in turn, may benefit from preventive and/or curative interventions. In clinical practice, the importance of cascade genetic testing is emphasized during genetic counseling, and probands are encouraged to inform relatives of potential genetic risks.

Due to differences in healthcare systems, funding models, cultural attitudes, and legal frameworks, international approaches to cascade testing vary. In countries with universal healthcare, such as Italy, cascade testing is generally publicly funded, while in private insurance-based systems, such as the United States (US), coverage is inconsistent (Caswell-Jin et al. 2019; Costanzo et al. 2025; Kurian and Katz 2020; Ongaro et al. 2025). Some countries have well-established national protocols for specific conditions, while in others, cascade testing is offered less systematically, depending on individual clinicians or regional resources (Chiang et al. 2024). In most jurisdictions, privacy laws locate sole responsibility for disclosing genetic risk information to at-risk relatives with the proband, but in some, healthcare professionals (HCPs) are allowed to directly contact probands’ at-risk family members (Dheensa et al. 2016; Ehrencrona et al. 2025).

Factors such as family cohesion and genetic literacy further shape intra-familial communication of genetic risk. Families characterized by stronger cohesion are generally more open to discussions and more supportive of cascade communication, whereas weaker cohesion has been associated with incomplete or selective disclosure (Forrest et al. 2003; Wiseman et al. 2010). Likewise, higher levels of genetic literacy—defined as a multidimensional construct encompassing awareness, skills, and knowledge—can enhance individuals’ confidence in engaging relatives with genetic information.

Despite its clinical utility, cascade testing remains underutilized, with uptake documented below 50%.largely due to reliance on proband-mediated disclosure and challenges in intra-familial communication (Afaya et al. 2024; Godino et al. 2021; Levine et al. 2024). These limitations have sparked a debate on whether responsibility for disclosure should rest with probands alone or be shared with HCPs (Samuel et al. 2017).

To date, research on the disclosure and communication of genetic risk information within families has mostly examined the views of patients (Frey et al. 2022) or the general public (Godino et al. 2025b). Far less is known about the personal preferences of HCPs regardingreceiving and communicating personal genetic risk information. Understanding these perspectives may contribute to a broader view of how HCPs perceive genetic risk and approach communication within clinical contexts, although the relationship with actual professional behavior remains to be further explored (Frank et al. 2013; Khubchandani et al. 2024).To our knowledge, no similar studies have been conducted among nurses, despite their central educational role towards patients and family members (Kwame and Petrucka 2021), including in the context of genetics (Dante et al. 2025; Godino et al. 2024, 2025a; Laaksonen et al. 2022). Furthermore, no studies have investigated nurses’ wish to be informed about familial genetic risks, how they would prefer this information to be communicated, and how such preferences might be influenced by their genetic literacy, specifically in its awareness dimension, nor their views on moral responsibility for cascade communication. This study attempts to fill in these gaps in the nursing literature, especially considering the growing relevance of genetic diseases and the importance of their early identification in improving prevention and health outcomes (Khoury and Dotson 2021; Modell et al. 2025).

The study aimed to (a) explore Italian nurses’ preferences and views regarding the disclosure and sharing of genetic risk information within families, with attention to their genetic literacy and views on responsibility, and (b) compare these perspectives with those of Italian laypeople.

In this study, genetic literacy was examined in its awareness dimension, referring to the recognition of the clinical relevance and applications of genetics in healthcare and its implications for family communication (Hurle et al. 2013; Little et al. 2022), and responsibility, particularly moral responsibility, was conceptualized as an awareness of the consequences of genetic knowledge and the moral agency to act upon it (Leefmann et al. 2017; Prainsack et al. 2016).

## Methods

### Design

This study is part of a broader research project that investigated the attitudes of the Italian population about the communication of genetic risk information (*reference blinded for review*) using a cross-sectional online survey design. Specifically, a nested analysis focusing on a subgroup of survey participants following the Checklist for Reporting Knowledge, Attitude, and Practice Studies (ChecKAP) (Zarei et al. 2024) (Supplementary Material: File S1) was performed between late 2024 and early 2025. Although the Reflexive Thematic Analysis Reporting Guidelines (Braun and Clarke 2024) is specifically designed for purely qualitative studies, we reviewed its recommendations and incorporated relevant elements to ensure transparency and rigor in performing and reporting the qualitative component of this nested study.

### Setting and sample

Participants for the online survey were recruited through multiple digital channels, including Facebook© (personal pages and interest groups), Instagram©, LinkedIn©, and the personal mailing lists of the authors and their professional networks at the national level. The advertisement posts briefly described the study as an investigation into public views on the communication of genetic risk information and included a direct link to the anonymous questionnaire, which was hosted on the Microsoft Forms platform. The survey was open from November 24, 2024, to February 7, 2025. There were eligible citizens satisfying the following inclusion criteria: (1) being at least 18 years old, (2) having no personal or family history of hereditary disease (regardless of previous genetic testing status), and (3) being able to provide informed consent.

During data collection, a considerable number of respondents self-identified as HCPs. Although the survey was not originally intended to target this population, the absence of existing evidence and the potential relevance of HCPs’ preferences with regard to their influence on patients and their families (Frank et al. 2013; Khubchandani et al. 2024), prompted us to conduct a dedicated analysis. Specifically, although nurses were not the intended target population, their unexpectedly high participation provided a unique opportunity to explore their personal, rather than professional, preferences on intra-familial genetic risk communication, and to compare them with those of Italian laypeople (Godino et al. 2025d; 2026c). As this was an unplanned sub-analysis, no a priori sample size calculation was performed; the final sample size corresponded to the number of HCPs respondents who met the inclusion criteria.

### Data collection instrument

The online survey was developed based on a systematic review of the literature, which revealed the absence of previously validated instruments addressing the study focus (Godino et al. 2025b). Consequently, survey items were developed to reflect key domains repeatedly highlighted in the literature as central to the communication of genetic risk information, including genetics literacy (awareness dimension), personal and family experiences, moral responsibilities, communication preferences, and potential psychological implications.

The instrument was structured into eight main sections covering: (1) genetic literacy (awareness dimension), (2) personal and family experiences with genetics, (3) perceived moral responsibilities, (4) communication and disclosure preferences, (5) family functioning, (6) potential psychological implications, (7) attitudes toward being informed of genetic risk, and (8) socio-demographic information. A conditional logic structure was implemented to tailor the flow of questions according to participants’ previous responses. The questionnaire is available in Supplementary Material (File S2).

The genetic literacy (awareness dimension) section included four items assessing participants’ awareness of both more recent (determining how a disease should be managed following diagnosis; determining which medication may or may not be effective for an individual) and more established applications of genetics (determining the risk or probability of developing a specific disease; determining the probability of transmitting a hereditary disease to offspring).

Because family functioning has also been identified as a key factor shaping intra-familial communication of genetic risk (Forrest et al. 2003; Wiseman et al. 2010), it was assessed through the Italian adaptation of the SCORE-15 (Systemic Clinical Outcome and Routine Evaluation) scale (Paolini and Schepisi 2020). The validated 15-item self-report tool covers strengths/adaptability, emotional strain, and intra-family communication (α = 0.80), with lower total scores indicating healthier family dynamics.

As part of the survey, participants were presented with three hypothetical scenarios (regarding pathogenic variants associated with Hereditary Breast and Ovarian Cancer, early-onset Alzheimer’s disease, or Cystic Fibrosis identified in a second-degree relative) and asked whether they would want to be informed of the risk (25% in each scenario) of having inherited the variant Participants who indicated a preference not to be informed of a genetic risk in the family for all three scenarios were given the opportunity to elaborate on the reasons for their choice in an open-ended question. Finally, participants had the option to provide further remarks at the end of the questionnaire.

A preliminary pilot phase was carried out to evaluate clarity and comprehensibility, resulting in adjustments before large-scale dissemination (Godino et al. 2025c).

### Data analysis

The overall study was conducted within a pragmatic paradigm, which emphasizes the use of methods best suited to address the research questions and generate findings of practical relevance. This nested study reflected the overall study: according to its aims, data on nurses were focused and compared with (a) lay public to highlight similarities and differences in genetic literacy, responsibility, and communication preferences and (b) with non-nurse HCPs.

Close-ended items were analysed using IBM SPSS (Version 28). Descriptive statistics, including means, standard deviations, ranges, and frequencies, were calculated to summarize participants' demographics, family cohesion, and genetic literacy. Independent samples t-tests were conducted to compare genetic literacy scores between nurses and non-nurse HCPs and nurses and laypublic. Chi-square tests were utilized to compare preferences for communication sources and information disclosure scenarios among participants. Linear regression analyses were performed to examine associations between genetic literacy and participants’ age and educational level. To evaluate associations between moral responsibility beliefs and participant characteristics (age, gender, parental status, genetic literacy), binary logistic regression models were employed. Results are presented as odds ratios (ORs) with 95% confidence intervals (CIs). Two-tailed *p*-values < 0.05 were considered statistically significant.

Open-ended responses were analysed using Reflexive Thematic Analysis, following Braun and Clarke’s six-phase framework (Braun and Clarke 2019). This approach was selected because it aligns with the exploratory aims of the study and acknowledges the active role of the researchers in generating themes, rather than relying on a predetermined framework. Initially, researchers familiarized themselves with the open-ended responses by repeatedly reading the data. The analysis proceeded inductively, with codes generated through constant comparison. During the coding stage, segments of text were systematically reviewed, interpreted, and organized into emerging categories. Subsequent steps involved clustering these codes into broader themes, refining them to eliminate redundancy, and examining their coherence within the entire dataset. Themes were then clearly labelled and described to capture their conceptual meaning, and the final stage integrated them into an overall interpretive account.

Although Reflexive Thematic Analysis does not require formal inter-coder reliability, two researchers (LG and VG) independently coded the data and engaged in iterative discussions to compare and refine interpretations. Divergences were openly debated and resolved with input from a third researcher (LB), enhancing both analytical depth and credibility.

The adequacy of the qualitative dataset was evaluated using the concept of information power (Malterud et al. 2016), considering the specificity of the participant group, the narrow study aim, and the relevance of the open-ended responses to the research question. While traditional notions of data saturation are less applicable to Reflexive Thematic Analysis, the information power of the dataset was deemed sufficient to generate meaningful insights (Malterud et al. 2016).

In presenting the results, representative quotations were exacted and used to support each theme. To aid contextualization, excerpts were accompanied by participants’ identifiers, gender, age, and genetic literacy (awareness dimension) score.

### Reflexivity and positionality

In line with recommendations for transparency in qualitative research (Jacobson and Mustafa 2019; Wainstein et al. 2023), the research team engaged in reflexive practices throughout the study. The authors’ positionalities (nursing, genetic counselling, bioethics, clinical genetics, and qualitative methodology) shaped the design, conduct, and interpretation of the findings. The first author is a genetic nurse and registered genetic counsellor; the team also included senior academics, experienced clinicians, and a master’s student trained and supervised during analysis. While anonymity of the online questionnaire reduced direct power imbalances, reflexive discussions were held to consider how the researchers’ professional and personal perspectives influenced the study process.

### Ethical considerations

The study adhered to the ethical standards of the Declaration of Helsinki and received approval from the Ethics Committee of the University of Bologna, Italy, on September 30, 2024, with formal authorization issued on October 11, 2024 (approval number 0313516).

## Results

**Fig. 1:**
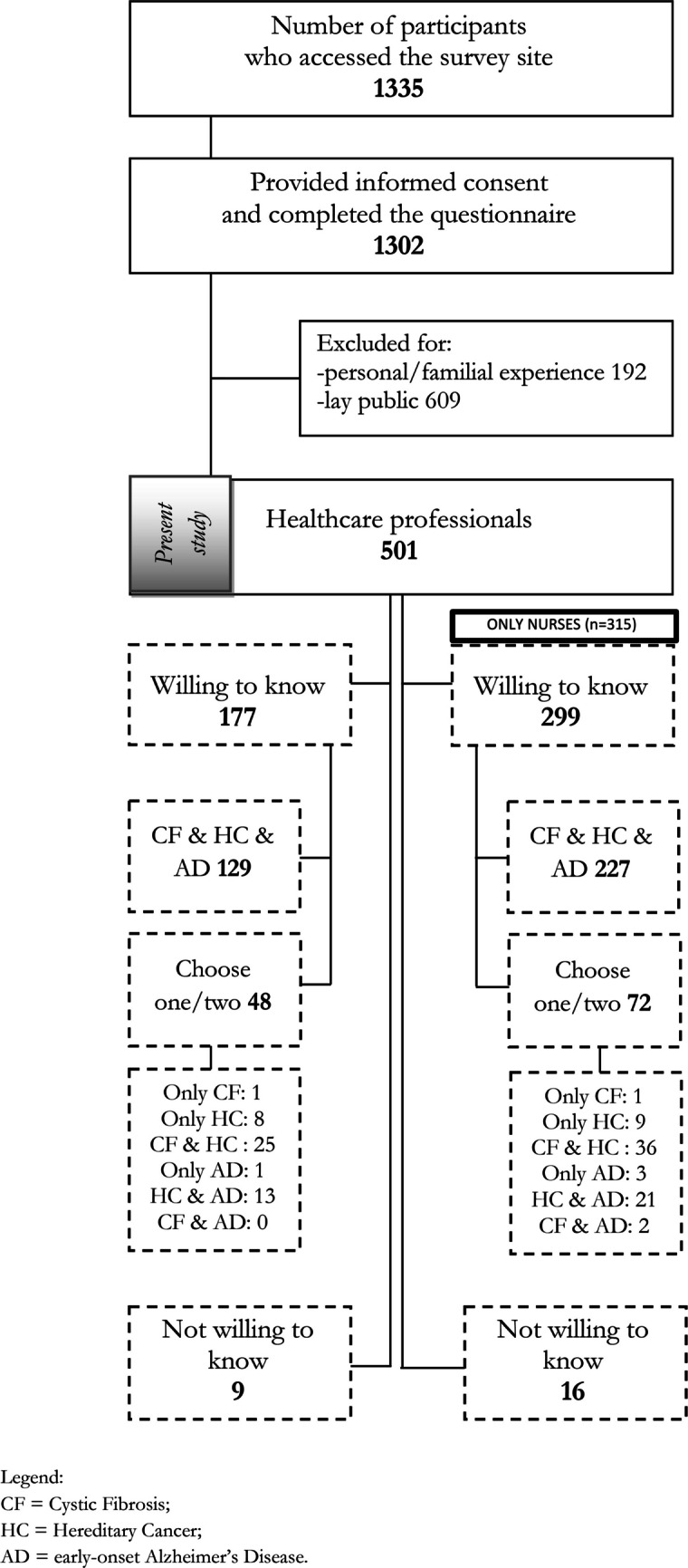
Flowchart of participant recruitment and selection

The diagram of survey participants is shown in Fig. 1. Of the 1,335 individuals who accessed the survey, 1,302 provided consent and completed it. After excluding 192 respondents with a personal or family history of hereditary conditions and 609 lay individuals, the final sample included 501 HCPs. The lay population (*n* = 609), derived from the same survey and previously described elsewhere (*reference blinded for review*), was used for comparative analyses. The characteristics of both HCPs (nurse and non-nurse participants) and lay participants are presented in Table 1.

**Table 1:** Characteristics of the study participants

		Lay public# (*N* = 609)		Nurse participants (*n* = 315)		Non-nurse participants (*n* = 185)		*p*-value
Variables				Mean	SD	Mean	SD	
Age, years		39.8	14.8	43.4	11.5	42.7	11.0	0.501
		n	%	n	%	n	%	
Gender*								
	*Male*	181	29.7	58	18.5	28	15.1	0.391
	*Female*	417	68.5	256	81.5	157	84.9	
Marital status								
	*Single*	227	37.3	68	21.6	43	23.1	0.458
	*Married*	211	34.6	135	42.9	89	47.8	
	*Cohabiting*	144	23.6	89	28.3	47	25.3	
	*Divorced*	21	3.4	19	6.0	6	3.2	
	*Widowed*	6	1.0	4	1.3	1	0.5	
Children								
	*No*	348	57.1	154	48.9	85	45.7	0.518
	*Yes*	261	42.9	161	51.1	101	54.3	
Highest level of education								
	*Middle School Diploma*	50	8.2	2	0.6	2	1.1	0.134
	*High School Diploma*	260	42.8	42	13.3	18	9.7	
	*Bachelor's Degree*	210	34.5	165	52.4	81	43.5	
	*Postgraduate Specialization*	64	10.5	82	26.0	78	41.9	
	*Doctorate (PhD)*	24	3.9	23	7.7	7	3.8	
Healthcare professionals								-
	*Audiometric technician*					1	0.2	
	*Biologist*					11	2.2	
	*Biomedical laboratory technician*					14	2.8	
	*Dental Hygienist*					1	0.2	
	*Dietitian*					2	0.4	
	*Healthcare assistant*					28	5.6	
	*Medical doctor*					63	12.6	
	*Midwife*					13	2.6	
	*Neuro and Psychomotor Therapist of Developmental Age*					1	0.2	
	*Neurophysiopathology technician*					2	0.4	
	*Nurse*			315	62.9			
	*Odontologist*					2	0.4	
	*Orthotic-Ophthalmologic assistant*					2	0.4	
	*Pharmacist*					7	1.4	
	*Phisiotherapist*					19	3.8	
	*Professional educator*					4	0.8	
	*Psychiatric rehabilitation technician*					2	0.4	
	*Psychologist*					7	1.4	
	*Speech therapist*					3	0.6	
	*Veterinarian*					2	0.4	
	*X-ray technician*					1	0.2	
Religious orientation								
	*Believer (Practing/Non-practing)*	401	65.8	220	69.8	117	37.1	0.116
	*Non-Believer/Agnostic*	208	34.2	95	30.2	69	37.1	
Current area of residence								
	*Northern Italy*	345	56.7	218	69.2	163	87.6	< 0.001
	*Central Italy*	118	19.4	75	23.8	11	5.9	
	*Souther Italy and Islands*	136	22.3	20	6.3	11	5.9	
	*Abroad*	10	1.6	2	0.6	1	0.5	
		Mean	SD	Mean	SD	Mean	SD	
Family relationship (SCORE-15), score 1–5**		2.0	0.6	2.0	0.6	2.0	0.6	0.737
Genetic literacy (awareness), score 0–4***		1.9	1.7	2.6	1.6	3.0	1.4	< 0.001

Legend: *SD* Standard deviation, *HCP* Healthcare professional, *SCORE-15* Systemic clinical outcome and routine evaluation scale

^#^ Godino et al., 2026c

^*^Two respondents who selected 'prefer not to disclose' for sex were excluded and treated as missing data

^**^ The total score ranges from 1 (optimal family functioning) to 5 (severe family dysfunction)

^***^ The total score ranges from 0 (no awareness of any clinical applications of genetics) to 4 (awareness of all four applications)

Mean and standard deviation (SD) are reported only for continuous variables (age, SCORE-15, and genetic literacy)

In line with the study aims, the main analyses focused on Italian nurses’ preferences and views regarding the disclosure and sharing of genetic risk information within families, including genetic literacy and views on responsibility (Tables 2, 3, 4, 5 and 6). Comparisons between nurses and the Italian laypeople are presented in Table 7,while comparisons with non-nurse HCPs are reported in the Supplementary Material.

**Table 2:** Comparisons of groups with different "Wanting to Know" responses among nurses (*N* = 315)

		Wanting to be informed about at least one condition versus none (*N* = 315)					Wanting to be informed about three conditions versus one or two conditions (*N* = 299)				
		None		At least one			1 or 2 conditions		All 3 conditions		
		(*n* = 16)		(*n* = 299)			(*n* = 72)		(*n* = 227)		
Characteristics of the study participants											
		Mean	SD	Mean	SD	*p*-value	Mean	SD	Mean	SD	p-value
Age		49.8	12.2	43.0	11.3	**0.022**	43.6	11.7	42.8	11.2	0.608
		n	%	n	%		n	%	n	%	
Gender*											
	*Male*	4	25.0	54	18.1	0.508	13	18.3	41	17.1	1.000
	*Female*	12	75.0	244	81.9		58	81.7	186	81.9	
Marital status											
	*Single*	4	25.0	64	21.4	**0.018**	12	16.7	52	22.9	0.539
	*Married*	6	37.5	129	43.1		32	44.4	97	42.7	
	*Cohabiting*	2	12.5	87	43.1		21	29.2	66	29.1	
	*Divorced*	4	25.0	15	5.0		6	8.3	9	4.0	
	*Widowed*	0	0.0	4	1.3		1	1.4	3	1.3	
Children											
	*No*	7	43.8	147	49.2	0.799	32	44.4	115	50.7	0.417
	*Yes*	9	56.3	152	50.8		40	55.6	112	49.3	
Highest level of education											
	*Middle School Diploma*	1	6.3	1	0.3	0.300	0	0.0	1	0.4	0.396
	*High School Diploma*	3	18.8	39	13.0		10	13.9	29	12.8	
	*Bachelor's Degree*	7	43.8	158	52.8		35	48.6	123	54.2	
	*Postgraduate Specialization*	4	25.0	78	26.1		19	26.4	59	26.0	
	*Doctorate (PhD)*	1	6.3	23	7.7		8	11.1	15	6.6	
Religious orientation											
	*Believer (Practing/Non-practing)*	9	56.3	211	70.6	0.265	57	79.2	154	67.8	0.075
	*Non-Believer/Agnostic*	7	43.8	88	29.4		15	20.8	73	32.2	
Current area of residence											
	*Northern Italy*	11	68.8	207	69.2	0.990	53	73.6	154	67.8	0.310
	*Central Italy*	4	25.0	71	23.7		12	16.7	59	26.0	
	*Souther Italy and Islands*	1	6.3	19	6.4		6	8.3	13	5.7	
	*Abroad*	0	0.0	2	0.7		1	1.4	1	0.4	
		Mean	SD	Mean	SD		Mean	SD	Mean	SD	
Genetic literacy (awareness), score 1–5**		1.8	1.6	2.6	1.6	0.057	2.8	1.5	2.6	1.7	0.385
Family relationship (SCORE-15), score 0–4***		2.0	0.5	2.0	0.6	0.959	2.1	0.6	2.0	0.6	0.197

Legend: *SD* Standard deviation; *SCORE-15* Systemic clinical outcome and routine evaluation scale

^*^One respondent who selected 'prefer not to disclose' for sex were excluded and treated as missing data

^**^ The total score ranges from 1 (optimal family functioning) to 5 (severe family dysfunction)

^***^ The total score ranges from 0 (no awareness of any clinical applications of genetics) to 4 (awareness of all four applications)

**Table 3:** Cross-scenario themes underlying nurse participants’ desire to know genetic risk information, with scenario-specific expressions

Theme	Scenario-specific expressions	Illustrative quotes
Genetic information as a basis for action	**CF:** Reproductive screening and carrier awareness to inform family planning decisions	*“For reproductive risks linked to cystic fibrosis”*
	**HC:** Prevention, surveillance, and lifestyle changes to reduce or manage disease risk	*“To take preventive measures, if possible."* *“To make better lifestyle choices."*
	**AD:** Early recognition of symptoms and behavioral adjustments, where possible, to manage future disease progression	*“To avoid being caught off guard by the first signs of the disease and take preventive action by correcting or changing certain behaviors.”*
Genetic information as support for responsible decisions toward oneself and one’s family	**CF:** Avoiding transmission to offspring and making informed reproductive choices	*“For family planning and check-ups "* *"To avoid having children if the result is positive."*
	**HC:** Making informed health decisions and protecting oneself and family members through preventive care	*"To have the option to decide whether or not to have the test and make informed choices."* *"To enhance preventive screening."*
	**AD:** Informing children and reducing the emotional or caregiving burden on relatives	*“To keep my children informed and support their family planning decisions ."*
Genetic information as preparation for the future	**CF:** Planning future parenthood and reproductive decisions	*“To make informed choices if my partner is also a healthy carrier, and thus avoid the risk of having a child affected by the disease”*
	**HC:** Anticipating disease risk and organizing long-term monitoring	*"For family planning and preventive check-ups."*
	**AD:** Psychological preparation, life planning, and managing future loss of autonomy	*" To prepare myself psychologically in case it should it happen to me."* *“To plan my future and be prepared for the possible onset of the disease and its long-term management."*

**Table 4:** Preferences for the source, situation, and method of receiving genetic risk information among nurse participants (*N* = 315)

Items	n	%
Item 13. In your opinion, who has the moral responsibility to inform you?		
*All (self, relatives, healthcare professionals)*	75	25.1
*Only self*	3	1.0
*Only relatives*	20	6.7
*Only healthcare professionals*	49	16.4
*Both relatives and healthcare professionals*	117	39.1
*Both self and relatives*	11	3.7
*Both self and healthcare professionals*	21	7.0
*No one/Not known*	3	1.0
Item 15 – Preferred family member to receive the information from*:		
A family member who has already taken the genetic test	116	54.0
A family member with whom I have a close relationship	33	15.3
A family member I am in regular contact with	8	3.7
No particular preference	58	27.0
Item 18 – Preferred situation to be contacted and informed by a healthcare professional**:		
Always, regardless of my family's preference	180	69.2
If my family wants to inform me but has difficulty doing so, so they seek help from a healthcare professional	73	28.1
If my family does not want to inform me	7	2.7
Item 16 – Preferred method of receiving information from a family member***:		
Message (e.g. WhatsApp)	1	0.5
Email	1	0.5
Letter	2	0.9
Phone call	5	2.3
Video call	0	0.0
In person	204	95.8
Item 20 – Preferred method of receiving information from a healthcare professional****:		
Message (e.g. WhatsApp)	1	0.4
Email	11	4.5
Letter	0	0.0
Phone call	11	4.5
Video call	1	0.4
In person	221	90.2

^*^ Only respondents who were open to be informed by family members were asked to give this preference (*n* = 215)

^**^ Only respondents who were open to be informed by a healthcare professional were asked to give this preference (*n* = 260)

^***^ Data missing for 2 participants

^****^ Data missing for 15 participants

**Table 5:** Binary logistic regression analyses of nurses’ perceived moral responsibility (*N* = 298)*

	Self (*n* = 298)		Relatives (*n* = 298)		Professionals (*n* = 298)	
Predictors	OR [95% CI]	*p-value*	OR [95% CI]	*p-value*	OR [95% CI]	*p-value*
Age	1.04 [1.02–1.07]	0.002	0.98 [0.96–1.01]	0.170	1.04 [1.00–1.08]	0.031
Gender	1.43 [0.74–2.76]	0.283	2.37 [1.27–4.45]	0.007	0.52 [0.17–1.54]	0.237
Knowledge	1.21 [1.04–1.42]	0.017	1.11 [0.95–1.31]	0.188	0.93 [0.74–1.17]	0.532
Having children	0.59 [0.34–1.03]	0.061	1.51 [0.84–2.73]	0.169	0.93 [0.41–2.08]	0.850

*OR* Odds ratio; *CI* Confidence interval. Self = 'I have the moral responsibility'; Relatives = 'My family members have the moral responsibility'; Professionals = 'Healthcare professionals have the moral responsibility'. Gender is coded as 0 = male (reference), 1 = female. Having children is coded as 0 = no (reference), 1 = yes; Knowing all conditions is coded as 0 = Knowing one or two conditions (reference), 1 = Knowing all three conditions

^*^Moral responsibility was assessed using Item 13 of the questionnaire

**Table 6:** Preferences for communication among nurse participants (*N* = 315)

		Strongly disagree	Disagree	Neither agree nor disagree	Agree	Strongly agree
Items		n (%)	n(%)	n(%)	n(%)	n(%)
(a) Preferences for communication from the perspective of a person who could receive the information						
Item 14	I would prefer to be informed by a family member	9 (3.0)	16 (5.4)	59 (19.7)	109(36.5)	106 (35.5)
Item 17	I would prefer to be contacted and informed by a healthcare professional	3 (1.0)	4 (1.3)	32 (10.7)	93 (31.1)	167 (55.9)
Item 21	I would prefer to be informed by a family member with whom I have no contact rather than not being informed at all	20 (6.7)	16 (5.4)	48 (16.1)	81 (27.1)	134 (44.8)
Item 22	I would prefer to be informed by a doctor/healthcare professional rather than not being informed at all	0 (0.0)	1 (0.3)	8 (2.7)	90 (30.1)	200(66.9)
Item 23	I would prefer to be informed by a doctor/healthcare professional rather than by a family member with whom I have no contact	3 (1.0)	10 (3.3)	58 (19.4)	86 (28.8)	142 (47.5)
Item 24	I would prefer to be informed first by a family member with whom I have a close relationship	13 (4.3)	19 (6.4)	98 (32.8)	93(31.1)	76(25.4)
(b) Preferences for communication from the perspective of the informant						
Item 27	If I was the first person in my family be diagnosed with a hereditary disease, I would personally inform my family members	2 (0.7)	7 (2.3)	18 (6.0)	92 (30.8)	180 (60.2)
Item 28*	If I were the first person in my family to be diagnosed with a hereditary disease, I would want healthcare professionals to inform my family members	18 (6.1)	36 (12.2)	98 (33.2)	84 (28.5)	59 (20.0)
Item 29*	If I were the first person in my family to be diagnosed with a hereditary disease, I would inform my family members with the help of health professionals	0 (0.0)	8 (2.7)	40 (13.6)	113 (38.3)	134 (45.4)

^*^Data missing for 4 participants

**Table 7:** Comparison of survey's main results between nurses and lay public

		Nurses (*N* = 315)		Lay public# (*N* = 609)		
Characteristics of the study participants		Mean	SD	Mean	SD	*p*-value
Age		43.4	11.5	39.8	14.8	< 0.001
Gender*		n	%	n	%	
	*Male*	58	18.5	181	29.7	< 0.001
	*Female*	256	81.5	417	68.5	
Results		n	%	n	%	
Genetic literacy (awareness), score 0–4**		2.6	1.6	1.9	1.7	< 0.001
Determining the risk or probability of developing a specific disease						
	No	77	24.4	250	41.1	< 0.001
	Yes	238	75.6	359	58.9	
Family relationship (SCORE-15), score 1–5***		Mean	SD	Mean	SD	
		2.0	0.6	2.0	0.6	1.000
Wanting to be informed about at least one condition versus none		n	%	n	%	
	None	16	5.1	44	7.2	0.260
	At least one	299	94.9	565	92.8	
Wanting to undergo genetic testing (if wanting to know at least one condition)****						
	**No**	20	2.2	68	4.4	0.005
	**Yes**	877	97.8	1461	95.6	
Wanting to be informed about three conditions versus one or two conditions						
	One or two conditions	72	24.0	124	21.9	0.500
	All three conditions	227	75.9	441	78.1	
Wanting to be informed about specific conditions*****						
	Cystic Fibrosis	39	29.8	52	25.2	0.631
	Hereditary Cancer	66	50.4	113	54.4	
	Early-onset Alzheimer’s disease	26	19.8	42	20.4	

Legend: *SD* Standard deviation; *SCORE-15* Systemic clinical outcome and routine evaluation scale

^#^ Godino et al., 2026c

^*^ The total score ranges from 1 (optimal family functioning) to 5 (severe family dysfunction)

^**^ The total score ranges from 0 (no awareness of any clinical applications of genetics) to 4 (awareness of all four applications)

^***^ Eleven respondents from lay population and two respondents from nurses who selected 'prefer not to disclose' for sex were excluded and treated as missing data

^****^ Numbers refer to the total number of responses across scenarios, not to the number of individual participants. Each participant contributed up to three responses (one per scenario)

^*****^ Numbers refer to the total responses across scenarios (*n* = 131 for nurses and *n* = 206 for lay respondents), not to the number of individual participants, as each participant could contribute more than one response

### Participants profileand genetic testing

Overall, the HCP sample had a mean age of 43.1 ± 11.3 years (range: 21–67), with a majority being female (82.4%) and nurses (62.9%). Specifically, nurses (*n* = 315) were mostly women, middle-aged, married, with children and held at least a bachelor’s degree; and self-reported as believers (Table 1) with no statistically significant differences compared to non-nurse HCPs. Nurse participants’ genetic literacy (awareness dimension) was moderate overall (2.5 ± 1.6, range 0–4), significant lower to that perceived by non-nurse HCPs (Table 1), and significantly higher compared with the lay public (mean = 1.9 ± 1.7, *p* < 0.001).

Specifically, 149 of 315 nurses (47.2%) scored 4, 74 (23.4%) scored 0, and the remainder were distributed across intermediate scores (score1: 3, 1.0%; score2: 45, 14.4%; score3: 44, 14.0%). Answers to four items assessing awareness of genetic testing applications as reported by nurses and non-nurses’ participants are detailed in the Supplementary Material (Table S1). Of note, participants’ awareness of more recent applications was lower than that of more established uses (52%/54.3% vs. 74.6%/75.6%; *p* < 0.001). Separate regression models showed that neither age (B = 0.001, SE = 0.008, β = 0.005, *p* = 0.934) nor educational attainment (B = 0.076, SE = 0.113, β = 0.038, *p* = 0.505) were associated with nurses’ genetic literacy scores.

### Wanting to know one’s genetic risk and have genetic testing

Of the 315 nurse participants, 299 (94.9%) wished to be informed about at least one condition, while 16 (5.1%) preferred not to receive any information. Among those open to information, 227 (75.9%) wanted to be informed in all three scenarios, and 72 (24.1%) selected one or two, most frequently Hereditary Cancer (HC, 50.4%), followed by Cystic Fibrosis (CF, 29.8%) and early-onset Alzheimer’s disease (early-onset AD, 19.8%). Detailed choice combinations are shown in Fig. 1, and display highly fragmented responses. Therefore, answers were categorised into two groups: (1) Wanting to be informed about one or two conditions and (2) Wanting to be informed about the three conditions.

Nurses who wished to be informed about at least one condition were significantly younger (43.0 ± 11.3 vs. 49.8 ± 12.2; *p* = 0.022) and showed higher scores in genetic literacy (awareness dimension), although not statistically significant (2.6 ± 1.6 vs. 1.8 ± 1.6; *p* = 0.057) (Table 2); these differences were not observed in non-nurse HCPs as a group (Supplementary Material Table S2).

Similarly, a high proportion of lay public participants expressed the desire to be informed (92.8%), with no significant difference compared to nurses (*p* = 0.260) (Table 7). However, nurses were slightly less likely than lay participants to want information across all three scenarios (75.9% vs 78.0%; *p* = 0.500). Among those choosing to be informed about one or two conditions, the proportion opting to learn about early-onset Alzheimer’s disease was almost identical in the two groups (19.8% vs 20.4%).

Open-ended responses (Why would you want to know?) offered insights into the drivers of participants’ decisions. Three broader themes were identified across the three scenarios: genetic information as a basis for action, as support for responsible decisions toward oneself and one’s family, and as a resource for future preparedness. These themes were expressed differently depending on the condition considered, with a stronger emphasis on reproductive responsibility in CF, prevention in HC, and life planning and emotional preparedness in early-onset AD (Table 3).

Finally, at least 96.4% of nurses who wished to be informed about a genetic risk in the family also indicated willingness to undergo genetic testing, compared with 94.2% of non-nurse HCPs (Supplementary Material Table S3) and 95.6% of lay participants, with a significant difference only between nurses and lay respondents (*p* = 0.005) (Table 7).

### Whose is the moral responsibility?

Regarding the moral responsibility of informing individuals about a genetic risk within the family, 117 of 299 (39.1%) stated that family members and HCPs share this responsibility, and 75 of 299 (25.1%) believed that it rests with everyone involved, namely themselves, family members, and HCPs. Other preferences were indicated by small numbers of respondents (e.g., only HCPs *n* = 49, 16.4%; only family members *n* = 20, 6.7%) (Table 4 and Table S4). Among non-nurse HCPs, 43.2% also believed that relatives and professionals share this moral duty, 27.9% attributed it only to professionals, and 9.0% only to relatives (Table S4). These distributions were broadly consistent with those observed among the lay population, who also tended to view moral responsibility as shared rather than individual, though with a slightly greater emphasis on professional responsibility (35.0%, 11.3%, and 8.0%, respectively).

When the association between moral responsibility of informing and various nurse participant characteristics (age, gender, parental status, genetic literacy) was evaluated using binary logistic regression models (Table 5), age was positively associated with agreement with the belief that informing is one’s own responsibility (*p* = 0.002) and female gender was positively associated with the belief that it is the responsibility of relatives (*p* = 0.007).

### Preferences about genetic risk communication from the recipient’s perspective

Overall, Items 14 to 24 explored communication preferences from the recipient’s perspective. Single item results are presented in Table 4 (Item 13,15,16,18,20) and Table 6 (Item 14,17,21–24) for nurse participants and in Supplementary Material for non-nurse HCPs (Table S5-S6).

Briefly, among nurse participants who wished to be informed (*n* = 299), 109 (36.5%) agreed and 106 (35.5%) strongly agreed that they would prefer to be informed by a family member (Item 14, Table 6). When asked about being informed by HCPs (Item 17, Table 6), 167 of 299 (55.9%) strongly agreed and 93 of 299 (31.1%) agreed.

A combined variable based on Items 14 and 17 showed that, among participants wanting to know at least one condition who expressed a clear preference between family members and HCPs as a source of information (168 of 299, 56.2%), 122 (72.6%) preferred family members and 46 (27.4%) preferred HCPs (Supplementary Material Table S7). Comparable distributions were observed among non-nurse HCPs (66.1% vs 33.9%) and among lay participants (61.2% vs 38.8%), although the difference between nurses and lay participants was statistically significant (*p* = 0.014).

Regarding family members as information source, most nurse respondents preferred to receive information from a family member who had already had a genetic test (54%, Item 15) while the presence of a close relationship with the informant family member seemed to be a less stringent preference. Indeed, most of these respondents expressed a preference to be informed by a family member with whom they had no contact rather than not being informed at all (27.1% agreed and 44.8% strongly agreed – Item 21).

Most nurse respondents were open to the possibility that genetic risk communication occur through an HCP. Specifically, they expressed a preference to receive genetic risk information from HCPs rather than from a family member with whom they had no contact (28.8% agreed and 47.5% strongly agreed – Item 23) or than not being informed at all (30.1% agreed and 66.9% strongly agreed – Item 22).

The preferred mode of receiving information was in person, whether the informant was a family member (95.8%, Item 16) or an HCP (90.2%), Item 20).

For all items, comparable proportions were observed among non-nurse HCPs (Supplementary Material) and lay respondents (Godino et al., 2026c).

### Preferences about genetic risk communication from the informant’s perspective

Items 26 to 29 explored communication preferences from the informant’s perspective.

When participants were asked to indicate which family members should be informed (Item 26), 171 of 299 (57.2%) nurses stated all relatives, including those with whom participants had no regular contact, 88 of 299 (29.4%) only first-degree relatives, 36 of 299 (12.0%) only those they regularly interact with and 4 of 299 (1.3%) reported none (Supplementary Material Table S8).

Single item results for Item 27–29 are reported in Table 6. Briefly, most nurse participants (272 of 299, 91.0%) agreed they would personally inform family members (Item 27), and 143 of 299 nurses (47.8%) agreed having HCPs take on this role (Item 28). A combined variable based on Items 27 and 28 showed that, among the 216 of 299 (72.2%) nurses who expressed a clear ranking, 187 (86.6%) preferred to inform relatives personally, while 29 (13.4%) preferred professional disclosure (Supplementary Material Table S9). However, collaboration with HCPs in genetic risk communication emerged as an important consideration as most nurse participants (247 of 299, 83.7%) agreed that they would prefer to inform relatives with professional support (Item 29) (Table 6).

For all items, comparable proportions were observed among non-nurse HCPs (Supplementary Material) and lay respondents (Godino et al. 2026c).

### Family cohesion and communication-related preferences

Family cohesion, as assessed by the SCORE-15, was comparable across nurses, non-nurse HCPs, and lay participants, with all groups showing moderate relational functioning (mean 2.0 ± 0.6; range: 1–4; Tables 1 and 7). No significant differences emerged between nurses and non-nurse HCPs (*p* = 0.737), nor between HCPs and lay participants (*p* = 1.000). No significant associations were found between family cohesion and willingness to be informed, attitudes toward moral responsibility, or preferences for genetic risk communication (all *p* > 0.05).

### Preferring not to receive information about a genetic risk in the family

Among the 16 nurse participants who preferred not to receive any information, several reasons emerged from the open-ended response. These reasons clustered around three major thematic categories: 1) Worry about anxiety and emotional distress in oneself; 2) Protection against psychological harm; 3) Uncertainty and scepticism about preventive medicine.

Some participants were concerned about the potential emotional burden associated with knowing one’s genetic risk. They feared that such knowledge could trigger panic or anxiety for themselves:

“*It would definitely affect me; I would be very worried. It could compromise my peace of mind and my quality of life.*” (ID 178, Female, 43y, Genetic literacy score: 3, Nurse)

“* The seriousness of the disease would cause me too anxiety in my everyday life.*” (ID 341, Male, 26y, Genetic literacy score: 4, Nurse)

Others consciously preferred to focus on the present and avoid potentially distressing information. This protective stance was often framed as a strategy to maintain serenity and mental well-being:

“*I want to live in the present without worrying about the future.*” (ID 79, Female, 56y, Genetic literacy score: 4, Nurse)

“*If the risk isn’t high, I’d rather avoid unnecessary worries.*” (ID 809, Female, 43y, Genetic literacy score: 2, Nurse)

Finally, some participants expressed distrust in the medicalization of risk and questioned the utility of predictive testing. Their comments often conveyed a sense of scepticism:

“*I follow other schools of scientific thought that I consider equally valid”* (ID 548, Female, 60y, Genetic literacy score: 0, Nurse)

“*I no longer trust the national health system. Enough!!*” (ID 863, Female, 63y, Genetic literacy score: 3, Nurse)

## Discussion

### Participants' profile, genetic literacy, and genetic testing

This study investigated the genetic literacy (awareness dimension), preferences, perceived moral responsibility and views of Italian nurses who answered a survey on intrafamilial communication of genetic risk information and compared them with those of the lay public. This study also provides a broad comparison of nurses’ perspectives with those of non-nurse HCPs, given that the group of non-nurse HCPs was extremely diverse in its composition.

The socio-demographic features of the nurses who participated in the survey are consistent with those of Italian nurses generally, as described in national reports, with the exception of age and geographic location. Indeed, our participants were slightly younger than the national mean, which may be explained by a selection bias associated with the fact that the survey was disseminated online, and mostly from the north and center of Italy. Nurses reported modest genetic literacy (mean 2.5/4), which was not associated with age or level of education. Their genetic literacy was significantly lower than that reported by non-nurse HCPs in our study. In addition, there was a significant difference between nurses’ awareness of established applications of genetic testing and uses associated with precision medicine, suggesting that literacy is most lacking regarding more recent applications of genetic testing in clinical practice. The fact that almost 20% of the nurses scored 0 also deserves attention, as it appears to contrast with the results of earlier studies on Italian nurses, which suggested adequate understanding of basic genetic concepts (Godino et al. 2013, 2026b). The apparent contradiction may be explained by the focus of our instrument: while the prior surveys assessed knowledge of fundamental concepts, the items in this questionnaire targeted awareness of clinical applications. Perhaps not surprisingly, around 8% of the nurses in Godino et al. (Godino et al. 2013) reported that they were very confident when applying genetics in daily practice, while most described themselves as minimally (44.9% vs. 42.8%) or little confident (26.8% vs. 33.9%), and 11–15% reported no confidence at all. These findings may suggest that nurses experience difficulties in transferring theoretical knowledge to the clinical setting, even if they worked in specialties where genetics is most relevant, such as oncology, pediatrics, and cardiology. This aligns with international findings showing that, although nurses generally acknowledge the importance of genetics, they often lack the skills to integrate it into practice (Carpenter-Clawson et al. 2023; Laaksonen et al. 2022). Thus, the findings observed here may reflect a persistent gap between theory and current practice. This is also consistent with evidence from a recent scoping review, which highlighted that genomic literacy among nurses, students, and faculty is generally low, and that, to date, educational interventions have had only a limited impact on practical competence (Dante et al. 2025).

This gap may be explained by structural and professional factors within the Italian context. At the system level, the genetic counsellor role has not yet been formally recognized within the National Health System, and educational pathways in genetics remain limited and heterogeneous (Crimi et al. 2020; Godino et al. 2025a). The role of genetic counsellors, and genetic nurses, thus remains to be clearly defined, and the associated competencies are neither formally recognized nor consistently integrated into clinical practice. Available evidence from regional data further suggests that the genetics workforce remains limited and unbalanced. Although there is no clear consensus on optimal staffing, previous estimates indicate an average of 0.3 full-time equivalent clinical geneticists and 0.7 genetic counsellors per 100,000 inhabitants, corresponding to a multidisciplinary model with a strong contribution of non-medical professionals (Dragojlovic et al. 2020). In contrast, data from the Emilia-Romagna region show 0.54 clinical geneticists per 100,000 inhabitants, but no formally recognized genetic counsellors or specialized genetic nurses, and only a marginal involvement of nurses in genetic counselling activities (Godino et al. 2024). This imbalance reflects a model that remains largely physician-centered, with limited integration of nursing roles within genetic services.

This situation is consistent with the wider European context, where genetic counselling is recognized as a key component of multidisciplinary care but remains inconsistently regulated across countries (Catapano et al. 2022). In parallel, the increasing demand for genetic services is not matched by a proportional growth in trained professionals, contributing to workforce and training gaps (Godino et al. 2024).

### Wanting to know one’s genetic risk and have genetic testing

Similar to the lay public, nurses’ genetic literacy in the awareness dimension did not significantly influence nurses’ willingness to be informed of genetic risk. Prior research instead has shown that higher genetic literacy is associated with greater engagement in genetic testing contexts, as demonstrated by participants in large-scale genetic studies reporting higher knowledge, familiarity, and skills compared to the lay population (Little et al. 2022).

Both nurses and non-nurse HCPs showed a high willingness to be informed about genetic risk, a tendency that was also evident among lay respondents. These findings are consistent with results reported by Middleton et al. (2016) in an international survey on hypothetical incidental findings, in which both the general population and non-genetics HCPs expressed a strong willingness to be informed.

However, nurses were slightly less likely than lay respondents to want information across all three scenarios, suggesting a somewhat more selective approach toward predictive information. Among those choosing to be informed about one or two conditions, the proportion opting to learn about early-onset Alzheimer’s disease was nearly identical between the two groups, indicating similar attitudes toward predictive information about incurable conditions.

When they were open to learning about a genetic risk in the family, nurses were more likely than lay respondents to express a willingness to undergo genetic testing, while no differences emerged between nurses and non-nurse HCPs. This result may be explained in part by nurses’ greater genetic literacy, specifically by their greater awareness of the role of genetic testing in establishing a person’s risk of developing a hereditary disease.

### Whose is the moral responsibility?

With respect to moral responsibility, most nurses considered that communication of genetic risk within families is a shared duty, which involves probands and HCPs (39.1%) or themselves, family members, and HCPs (25.1%), echoing the views of the non-nurse HCPs and general public, who similarly tended to view this responsibility as shared rather than individual.

The concept of responsibility is central to how individuals reflect on their moral obligations in sharing genetic risk (Prainsack et al. 2016). Among nurses, as among lay participants, this perspective may suggest an understanding of risk as embedded within social and relational contexts rather than confined to individual choice. In Italy, current practice places responsibility for disclosure on the proband, with HCPs adopting a supportive role (Varesco et al. 2024). However, as this study explored personal perspectives rather than clinical behavior, the extent to which these views translate into nurse professional practice remains unclear.

### Preferences about genetic risk communication from the recipient’s perspective

Our findings suggest that Italian nurses and laypeople tend to favor a shared model of genetic risk disclosure, potentially opening the door to a more proactive role for HCPs. This contrasts with the reported perspectives of genetics specialists, such as those in Belgium, where opinions vary from limiting responsibility to proband counseling to endorsing a formal duty to warn relatives (Phillips et al. 2021). Similar divisions have been reported elsewhere: some clinicians see responsibility as lying entirely with the proband (Akpinar and Ersoy 2014), while others emphasize the HCP’s role in encouraging disclosure (Mendes et al. 2024).

In our survey, nurses generally preferred to be informed of genetic risk by a family member—especially one who had already undergone testing or with whom they shared a close emotional bond—while at the same time valuing professional involvement as a complementary or alternative option. This balanced view may suggest that genetic communication is perceived not as an individual task, but as a collaborative process embedded within familial and professional relationships. Similar preferences were observed among non-nurse HCPs and lay participants, pointing toward a relational model of communication that values both empathy and accuracy (Di Pietro et al. 2020; Leenen et al. 2016; Wiens et al. 2013).

Most nurses preferred in-person communication—whether from relatives or professionals—indicating limited acceptance of impersonal or technology-mediated modes of information delivery. This finding aligns with results from the Italian general population (Godino et al. 2026c), which also emphasized the importance of direct, relational contact in sensitive genetic communication (Nazareth et al. 2021; Schmidlen et al. 2022; Siglen et al. 2022). These preferences highlight a tension between the ideal of family-mediated communication and the perceived need for professional support, underscoring the importance of relational trust and emotional closeness in mediating genetic disclosure.

### Preferences about genetic risk communication from the informant’s perspective

Most nurses expressed a clear preference for personally informing their relatives about a genetic risk, a view shared by non-nurse HCPs and lay participants. At the same time, nearly half valued the possibility of professional involvement as complementary support, indicating that disclosure is perceived not as an individual duty but as a shared process that benefits from collaboration. This relational model echoes the perspectives of the general population, who similarly emphasized the credibility and communicative clarity of HCPs while maintaining the central role of family members (Frey et al. 2022; Godino et al. 2025b).

Such findings may highlight nurses’ dual awareness—as both professionals and potential patients—of the emotional complexity surrounding the disclosure of genetic information. Like in previous studies, professional support was particularly valued when relatives might struggle to cope with distress or uncertainty (Henrikson et al. 2020; Marleen van den Heuvel et al. 2020; Menko et al. 2013). Overall, nurses appear to endorse a collaborative and ethically balanced approach that integrates empathy, accuracy, and shared moral responsibility in family communication about genetic risk (Dheensa et al. 2016; Gaff and Hodgson 2014; Mendes et al. 2016; Metcalfe et al. 2011).

### Family cohesion and communication-related preferences

Based on previous evidence showing that cascade genetic testing is influenced by degree of kinship (Costanzo et al. 2025; Di Pietro et al. 2020; Frey et al. 2022; Godino et al. 2026a; Menko et al. 2013; Trevisan et al. 2024), we hypothesized that family relational dynamics might also shape preferences regarding genetic risk communication. However, in the present study, family cohesion was not associated with willingness to be informed, attitudes toward moral responsibility, or preferences regarding whether, how, or by whom genetic risk information should be communicated. This finding may suggest that such preferences are shaped more by individual and ethical considerations than by perceived family relational functioning, although this result should be interpreted with caution according to the secondary analysis of our study, and the limited sample size.

### Preferring not to receive information about a genetic risk in the family

The open-ended responses of the 16 nurses who indicated a preference not to know about genetic risks highlighted concerns about emotional burdens, anticipatory anxiety, and skepticism toward preventive medicine. These self-protective stances mirror those reported by lay respondents (Godino et al. 2025d), and underscore that nurses, despite professional expertise, may feel the psychological weight of health-related information. Indeed, previous research has shown that nurses may underestimate personal risk, avoid preventive care, or rely on emotional detachment as a coping strategy (Holtzclaw et al. 2020; Obiebi et al. 2020).

Reluctance to receive genetic information often reflects psychosocial rather than cognitive factors. Anxiety and emotional distress are well-documented deterrents to engagement with genetic services (Jonsen et al. 1996; Keogh et al. 2017; Sarki et al. 2022), particularly when information concerns uncontrollable or incurable conditions (Hurley et al. 2012; Sweeny et al. 2010). In this light, avoidance can be interpreted as a protective response aimed at maintaining emotional stability and grounded in the ethical principle of non-maleficence and the right not to know (Andorno 2004), as also observed among individuals in the Italian general population who preferred not to receive genetic risk information (Godino et al. 2025d).

For some, not knowing may also represent an alternative form of care and moral responsibility, intended to protect relatives from distress rather than to reject preventive knowledge (Chivers Seymour et al. 2010; Cowley 2016). The expressed skepticism toward predictive medicine may reflect broader concerns about medicalization and the emotional costs of anticipating illness (Keenan et al. 2019; Khubchandani et al. 2024; Salardi 2014).

### Limitations

While this study provides valuable insights, some limitations should be considered. It was hypothetical in design, so responses may not fully reflect real-world behavior. The survey was not originally developed for HCPs, and little is known about the specific professional settings of participating nurses, although it is unlikely many worked in genetics, given the scarcity of such roles in Italy and the fact that a dedicated nursing specialty in medical genetics currently does not exist (Godino et al. 2024, 2025a).

Another limitation concerns the heterogeneity of the non-nurse HCP group, which included professionals from diverse backgrounds, including a proportion of medical doctors (*n* = 63). Detailed information on medical specialties and professional settings was not available, limiting the possibility to explore whether specific subgroups (e.g., those working in genetics-related fields) contributed differently to the observed results. Because of this variability, and the relatively small size of specific subgroups, it was not possible to conduct a more detailed comparative analysis across specific professional categories. This may have influenced the observed differences in genetic literacy between nurses and non-nurse HCPs.

Finally, the sample was self-selected and may overrepresent nurses with a stronger interest in genetics or ethical issues, while the online format may have excluded individuals with limited access to or familiarity with digital tools.

### Recommendations for further research

As the present study focused on nurses’ personal preferences rather than clinical behavior, future research should further explore how such perspectives may relate to professional practice. Future research should develop and validate instruments specifically designed to assess nurses’ genetic literacy and preferences regarding communication of genetic risk, rather than relying on tools created for the general population. Given the heterogeneity of the non-nurse HCP group in the present study, future investigations could also adopt more targeted sampling strategies to allow for profession-specific comparisons and a more granular understanding of attitudes across healthcare roles. Qualitative studies within the Italian nursing community are warranted to explore in depth the emotional and ethical dimensions of disclosure and non-disclosure choices, particularly through more in-depth data collection methods (e.g., interviews or dedicated narrative approaches), as responses to open-ended survey questions in the present study were often concise and did not allow for deeper exploration. In addition, intervention studies evaluating the impact of targeted educational programs on nurses’ ability to integrate genetics into practice are needed to address the persistent gap between theoretical knowledge and clinical application.

### Implications for policy and practice

Italian nurses’ limited familiarity with the clinical applications of genetic testing highlights shortcomings in current nursing education and practice. Strengthening training in genetics, particularly its clinical applications, appears crucial to support nurses’ role in health communication and to enhance their ability to engage patients and families in discussions about genetic risk.

Recent advances in genetic testing have expanded the capacity to identify genetic conditions and implement targeted prevention strategies. In this context, cascade genetic counselling and testing enable the identification of at-risk relatives and support timely preventive interventions. Strengthening nurses’ competencies is therefore crucial to support effective family communication and patient engagement in these processes.

## Conclusion

This study explored Italian nurses’ preferences and views regarding the disclosure and sharing of genetic risk information within families, focusing on their genetic literacy and responsibility, and compared these perspectives with those of laypeople. When asked to consider genetic risk communication within their own families, Italian nurses provided responses that mostly paralleled those of the Italian lay public. Their modest awareness of clinical applications of genetic testing suggests the need to strengthen genetics education in undergraduate and postgraduate nursing programs, ensuring adequate preparation for their role in supporting patients and families in communicating hereditary risk.

## Supplementary Information

Below is the link to the electronic supplementary material.

Supplementary file1 (DOCX 92 KB)

Supplementary Material 1

## Data Availability

The data that support the findings of this study are available from the corresponding author upon reasonable request.
