# Supplementary material for: Learning about the genetic risk in my family: Preferences of Italian nurses
Source: J Community Genet. 2026 May 11;17(3):60. doi: 10.1007/s12687-026-00892-w (PMC13161415; doi:10.1007/s12687-026-00892-w)
Supplement: Supplementary file 2 — Supplementary Material 1 [file 12687_2026_892_MOESM2_ESM.docx]

**Supplementary Material: Files and Tables**

This Supplementary Material is organized into two sections.

- Part 1 presents the supplementary Files (S1–S2), which include additional materials related to the study.
- Part 2 presents the supplementary Tables (S1–S9), which provide detailed statistical data and extended results complementing those reported in the main manuscript.

**Part 1**

**File S1: ChecKAP: KAP-Reporting Checklist**

|  | **ChecKAP: KAP-Reporting Checklist—Checklist of items that should be included in reports of *KAP studies*** | | **Pag.** |
| --- | --- | --- | --- |
| **Items** | **Item No#** | **ITEM RECOMMENDATION** |  |
| **TITLE**  **General Consideration:** the title should include the fact of reporting a CAP study, the subject of the KAP and possibly the population studied. | | |  |
| **Title** | **T1** | Describe the title identifying Knowledge, Attitude, and Practice or acronym (KAP) study | 1 |
| **ABSTRACT**  **General Consideration:** An informative abstract is a structured abstract with the headings of Objective, Method, Result and Conclusion. Typically, a well-structured abstract has a larger section for Results and Methods and a shorter section for Objective and Conclusion in terms of text length. Avoid including statements in the abstract that require references | | |  |
| **Abstract structure** | **A1** | Objective: Clearly state the purpose of the study in the objective, without including any background information or need of reasoning. | 3 |
|  | **A2** | Methods: Briefly describe the main methods used, including the type of study, how the sample was recruited, the tool you used to measure KAP | 3 |
|  | **A3** | Results: Present the most significant finding related to the objective, along with any other important results. Provide quantitative details, such as p-values or confidence intervals if applicable to your research. | 3 |
|  | **A4** | Conclusion: Summarize your conclusion without giving recommendations. | 3 |

| . **KEYWORDS**  **General Consideration:** The keyword should identify your study as a KAP study. The KAP’s subject, the population studied. | | | | | **Page** |
| --- | --- | --- | --- | --- | --- |
| **Key words** | **K1** | | | Make sure your keywords are listed in MeSH catalog | 3 |
| **INTRODUCTION**  **General Consideration:** Provide a comprehensive background, rationale, and objective(s) of the KAP study. In the Introduction, you are supposed to show the magnitude of the subject that KAP, the importance of a KAP study in relation to the subject of KAP, the existing knowledge and the gaps of knowledge, and how your objective addresses the gap. The background and introduction section serves as a foundation for the rest of your manuscript, setting the stage for the research you will present. It should engage readers, provide context, and make a clear case for the importance of your KAP study. In the background and introduction section of a manuscript about a Knowledge, Attitudes, and Practices (KAP) study, you should include the following items. Introduction normally  does not get a subheading and items can be accommodated in one or more paragraphs. | | | | | **Page** |
| **Introduction** | | **I1** | **Research Context and Rationale:** Begin by introducing the broader context of your study.  The magnitude of the problem (state incidence, prevalence, burden, or other descriptive  measures) show the magnitude of the problem, explains why the specific topic of the KAP study is important and relevant, providing a clear rationale for conducting the research. | | 5-6 |
|  |  | **I2** | **Statement of the Problem:** Clearly state the research problem or question that your KAP study aims to address. This should be a concise statement that outlines the specific area of  knowledge, attitudes, and practices that you will be investigating. | | 6 |
|  |  | **I3** | **Significance of the Study:** Explain the significance of your KAP study. Emphasize why understanding the KAP related to the topic is important and how it could contribute to  addressing the subject that the KAP is about. | | 6 |
|  |  | **I4** | **Literature Review:** Provide a brief review of relevant literature. Summarize key studies, findings, and theories related to the topic of your KAP study. This helps to contextualize your research within the existing body of knowledge and demonstrate how your study builds upon or  adds to the current understanding. | | 5-6 |
|  |  | **I5** | **Identify the gap:** Contextualize your research within the existing body of knowledge (the previous item) and demonstrate how your study builds upon or adds to the current  understanding. Light on the gaps in knowledge that your study is going to address. Try to be as bold as possible in presenting the gap and how your study is going to address the gap. (This part of the introduction is the part where you are going to convivence reviewer or evaluator that  your study merit novelty). | | 5-6 |
|  |  | **I6** | **The Objective of the study:** The last paragraph of the introduction should clearly outline the specific objectives of your KAP study. These objectives should outline what you intend to  achieve or uncover through your research. | | 6 |

| **METHODS**  **General Consideration:** In the method section of a manuscript for a Knowledge, Attitudes, and Practices (KAP) study should be as detailed as possible. The Method should have subheadings. | | | | **Page** |
| --- | --- | --- | --- | --- |
| **Methods^1^** | **Study design** | **M1** | Clearly describe the design of the KAP study (e.g., cross-sectional, longitudinal) and its appropriateness for the topic. | 6 |
|  | **Sampling Technique** | **M2** | Describe the sampling technique used and its rationale. | 6-7 |
|  | **Sample size** | **M3** | Report the sample size and provide justification for its adequacy.  Eligibility criteria for participants, including criteria at different levels in recruitment/sampling plan | 6-7  The sample size was not calculated a priori, as this study was based on a survey and aimed to include all respondents who completed the questionnaire during the data collection period. |
|  | **Data Collection Instrument** | **M4** | Describe the questionnaire or survey used to assess Knowledge, Attitudes, and Practices, including its development process. | 7-8 |
|  |  | **M5** | Is the KAP measurement tool considered a standard or researcher-developed?  In either case, referring to the validity and reliability of the tool is essential. | 7-8 |
|  |  | **M6** | **KAP score:**  Describe KAP score in detail (eg: Knowledge refers to responders’ level of knowledge about [topic/issue/problem].^2^ ; Attitude refers to responders’ feelings towards the subject. ^3^; Practice refers to the ways in which responder demonstrate their knowledge and attitude through their actions^4^. | 7 |
|  |  | **M7** | **Total KAP score**:  Description of total KAP score. It is used to rank the level of knowledge, attitude, and practice, and subsequent qualitative analysis was conducted to rank high, medium, and low scores. (e.g., How many scoring points were gained if a person answered all questions correctly?) | 7 |
|  |  | **M8** | Detail the data collection procedure, location, duration, and ethical considerations taken. Clearly describe the settings and locations where the data were collected | 7-10 |
|  | **Main Constructs**  **/Variables** | **M9** | Clearly define the variables of interest and describe the measurement methods used (e.g., Likert scales, open-ended questions).  Identify knowledge (Awareness) gaps, cultural beliefs (Attitude), or behavioral pattern (Practice) as main variables must be assessed | 7-8 |
|  | **Data analysis** | **M10** | Explain the statistical or qualitative analysis methods employed to analyze the KAP data. | 8-10 |
|  | **Ethical consideration** | **M11** | Describe the ethical considerations undertaken, including obtaining informed consent, ensuring participant confidentiality, and any necessary ethical approvals obtained. | 10 |

| **RESULTS**  ***General Consideration****: In the Results section of a manuscript for a Knowledge, Attitudes, and Practices (KAP) study, the following items of content should be included. (***Maybe all following items has not been mentioned in your manuscript)** but the content should somehow correspond what you explain in the method section. | | | **Page** |
| --- | --- | --- | --- |
| **Participant**  **Flow** | **R1** | Describe the flow of participants throughout the study, starting from recruitment and continuing through retention and any exclusions. Clearly detail the number of participants at each stage and any reasons for exclusion. | 10 |
| **Participant Characteristics** | **R2** | Provide detailed information about the study participants, including demographic, clinical, and social characteristics. This may encompass age, gender, educational level, socioeconomic  status, and any relevant medical history. | 10 |
| **Reporting of KAP’ Findings** | **R3** | **Knowledge Findings:** Present the results related to participants' knowledge in a structured manner. Highlight key findings, trends, and variations in knowledge levels among different participant groups, if applicable. . Support your description with tables or figures. (avoid very  simple figures).. | 10-11 |
|  | **R4** | **Attitudes Findings**: Similarly, present the results related to participants' attitudes in a clear and structured manner. Identify prevalent attitudes, variations, and any factors influencing attitudes within the participant population. Support your description with tables or figures. (avoid very simple figures). | 11-14 |
|  | **R5** | **Practices Findings:** Present the findings regarding participants' practices in a well-organized way. Describe common practices, deviations from recommended practices, and any factors affecting behavior. . Support your description with tables or figures. (avoid very simple  figures). | 11-14 |
| **Further Analysis -relating KAP to other variables.** | **R6** | **Correlation or other modeling:** If relevant, analyze and present any correlations or associations between knowledge, attitudes, and practices. Highlight significant relationships or  patterns that emerged from the data. Report the results quantitatively, using appropriate statistical measures such as mean, median, percentage, or frequency. Include confidence  intervals or p-values when applicable to provide a sense of the significance of the findings. | n/a |
|  | **R7** | **Subgroup Analyses**: If the study involved different participant subgroups (e.g., age groups,  gender), present separate findings for each subgroup and discuss any differences or similarities observed. | n/a |

| **DISCUSSION**  **General Consideration:** In the discussion section of a manuscript for a Knowledge, Attitudes, and Practices (KAP) study, the following items should be included. | | | **Page** |
| --- | --- | --- | --- |
|  | **D1** | Qualitatively report the main finding of your study in the first paragraph of the |  |
|  |  | discussion section (build a platform for flow of discussion). | 14-18 |
| **KAP results**  **interpretation**  **Scientific**  **justification** | **D2** | Explaining the strengths and weaknesses of the designed tool and comparing it with |  |
|  |  | other similar tools | n/a |
|  | **D3** | Sampling Bias: Explain any potential biases in the selection of participants that might affect the generalizability of your findings. | 18 |
|  | **D4** | Sample Size: Discuss whether the sample size was sufficient to draw meaningful conclusions and address the study’s objectives and representative of the target population, underscore how this enhances the reliability of your results. | 18 |
|  | **D5** | Self-Report Bias: Acknowledge the possibility that participants might not accurately report their knowledge, attitudes, or behaviors, leading to potential inaccuracies. | 18 |
|  | **D6** | Social Desirability Bias: Mention the chance that participants may provide responses they perceive as socially desirable, rather than their true beliefs or behaviors. | 18 |
|  | **D7** | Highlight the limitation of a cross-sectional study design pertaining to your KAP study. | 18 |
|  | **D8** | Recall Bias: Address the potential for participants to recall information inaccurately, especially when it comes to past behaviors or experiences. | n/a |
|  | **D9** | Cultural Context: state how cultural norms and values might have influenced responses and implication of this influence in your result and conclusion. | 14-18 |
| **Strength** | **D10** | Holistic Understanding: Highlight how the KAP study design allows for a comprehensive assessment of knowledge, attitudes, and practices, providing a well-rounded perspective. | 14-18 |
|  | **D11** | Real-world Application: Emphasize the practical implications of the study's findings, demonstrating how they could contribute to targeted interventions or policies. | 18 |
|  | **D12** | Structured Methodology: Explain how the study's structured approach to collecting and analyzing data contributes to the rigor and reliability of the findings. | 18 |
|  | **D13** | Quantitative and Qualitative Insights: If you used both quantitative and qualitative methods, highlight how this combination offers a more sensed understanding of the subject. | 14 and 18 |
|  | **D14** | Validation Measures If you used validated tools or instruments, mention how this adds validity to the study's outcomes. | 7 |
|  | **D15** | Report Limitations and Strength of the study at the end of the discussion. To specify the limitation as a headline is not necessary | 18 |
|  | **Conclusion** | |  |
| **Conclusion** | **D16** | Conclude the study’s result and its implications for practice and for further studies relevant to improve the short coming of your study or direct toward a gap that may exist. | 19 |

| **Acknowledgment and ethical consideration** | Page |
| --- | --- |
| **General Consideration:** Please state how the study addressed ethical conduct of the research, any disclosure that maybe need, if conflict of interest, if the study was approved by any scientific body, part of a student thesis, funded by a body, and etc.. | 2 |

**File S2**. Questionnaire English version

**QUESTIONNAIRE**

**Section 1: Genetic Conditions and Genetic Testing**

*Let’s start with a few general questions about hereditary diseases and genetic testing.*

1. Doctors can use genetic tests to care for their patients. Have you ever heard or read anything about this?

- Yes
- No

*If they answer “Yes”*

2. Have you ever heard or read that genetic tests can help doctors:

|  | Yes | No |
| --- | --- | --- |
| establish a person’s risk or probability of developing a specific disease? |  |  |
| decide how a disease should be managed after it is diagnosed in a person? |  |  |
| understand which drugs may or may not be effective for a person? |  |  |
| determine a person’s chance of passing on a hereditary disease to their children? |  |  |

**Section 2. Personal and Family Experience**

*Thank you for answering these general questions. Next are a few questions about your family experience. We understand that these questions may seem personal and sensitive. The reason we ask is to better understand people’s experiences about sharing genetic information within families*

3. Do you know whether there is a hereditary disease in your family?

- Yes
- No
- I do not know

4. If yes, what hereditary disease is it?

…………………………………………………………………………………………………

5. Who is affected? (multiple answers possible)

- You
- Other family member/s

6. Has a genetic test been performed for this disease in your family?

- Yes, I am the first in the family to have taken the test
- Yes, one (or more) of my family members has already taken the test, and I have too
- Yes, one (or more) of my family members has already taken the test, but I have not
- No
- I do not know

**Section 3: Hypothetical Scenarios of Hereditary Diseases**

*Thank you for answering the questions about your family experience. Now we will introduce three hypothetical situations involving* ***three different hereditary diseases****.*

*Please answer all the questions. There are no right or wrong answers. Select the option that best describes your point of view.*

Imagine that one of the following situations occurs in your family:

**Scenario 1**

One of your cousins is diagnosed with Cystic Fibrosis. Your aunt and uncle then discover that they both carry a genetic variant that causes this disease.

*Cystic Fibrosis is a genetic disease that primarily affects the lungs and the digestive system. Parents who carry one copy of a disease-causing genetic variant have no symptoms, but they can pass the variant on to their children. If both parents carry a variant, their children may develop the disease. Knowing their carrier status enables couples to make informed decisions about family planning. In Italy, about 1 in 25 people is a healthy carrier of a genetic variant that causes Cystic Fibrosis.*

**Scenario 2**

One of your aunts has ovarian cancer and has been found to carry a genetic variant that increases the risk of developing cancers such as breast, ovarian, and prostate cancer.

*People with this genetic variant are at high risk and can benefit from surveillance and prevention programs to facilitate early diagnosis and reduce their risk of developing cancer. In Italy, about 1 in 500 people has a genetic variant that increases the risk of developing these types of cancer.*

**Scenario 3**

An uncle of yours shows signs of Alzheimer's disease. Tests reveal that he carries a genetic variant that causes an early-onset form of the disease.

*Almost all individuals with this type of genetic variant develop the disease, and, currently, no preventive measures or medications can reduce the risk of this form of Alzheimer’s. This condition is very rare: the vast majority of Alzheimer's cases are* ***NOT*** *directly hereditary.*

Imagine that in these three scenarios, the uncle or aunt is your mother’s sibling, meaning you have a 25% probability (1 in 4 chance) of having inherited the genetic variant that runs on your mother’s side of the family.

**7** Would you want to know?

- Yes (*get to item 8*)
- No *(get to item 12)*

8 If your answer is yes, for which situation would you want to know?

Scenario 1 (Cystic Fibrosis) Yes/No

Scenario 2 (Cancer Predisposition) Yes/No

Scenario 3 (Alzheimer’s Disease) Yes/No

**9** Why would you want to know?

………………………………………………………………………………………………………………………………………………..……

**10** Would you want to take a genetic test to learn whether you inherited the genetic variant that runs in your family?

Scenario 1 (Cystic Fibrosis) Yes/No

Scenario 2 (Cancer Predisposition) Yes/No

Scenario 3 (Alzheimer’s Disease) Yes/No

**11** Why would you want to take the test? Why would you not? *(Get to next section*)

………………………………………………………………………………………………………………………………………………..……

**12** Why would you not want to know? (Get to items regarding Family Relationships) ………………………………………………………………………………………………………………………………………………..……

**Section 4: Moral Responsibility**

*Thank you for answering the questions about hypothetical situations. The next questions are about* ***who you think is morally responsible for informing you*** *about the genetic risk that runs in your family. Please keep in mind that when a genetic variant is found in a person with a disease, doctors usually recommend that they inform their family members, so they, in turn, can seek specialist advice to better understand their own risk. However, sharing this information with family members can be complicated or difficult. The result is that family members are often not informed.*

**13**: Who do you believe is morally responsible for informing you?

- - No one, the moral responsibility to inform myself is mine and I must be proactive about seeking the information.
  - Yes
  - No
  - I do not know

- - My family members
- Yes
- No
- I do not know

- - Doctors/Healthcare professionals
- Yes
- No
- I do not know

**13.a** Other (specifiy) ……………………………………………………………………………

**Section 5: Communication Preferences**

*Thank you for answering the questions o*n  *moral responsibility. The next questions are about* ***who you would prefer to receive the information from and how.***

*There are no right or wrong answers. Select the option that best describes your point of view.*

**14**. I would prefer to be informed by a family member.

| Strongly agree | Agree | Neither agree nor disagree | Disagree | Strongly disagree |
| --- | --- | --- | --- | --- |

*If answers “Strongly Agree” or “Agree” are given to the previous item*

**15** Which family member would you prefer to receive the information from?

- A family member who has already taken the genetic test
- A family member with whom I have a close relationship
- A family member I am in regular contact with
- No particular preference
- Other (specify) ……………………………………………………………………….

**16** How would you prefer to receive the information from your family member?

- In person
- By phone call
- By letter
- By email
- By text message (eg. WhatsApp)
- Other (specify)…………………………………………………………………

**17**. I would prefer to be contacted and informed by a healthcare professional

| Strongly agree | Agree | Neither agree nor disagree | Disagree | Strongly disagree |
| --- | --- | --- | --- | --- |

*If answers “Strongly Agree” or “Agree” are given to the previous item*

**18** In what situation would you prefer to be contacted and informed by a healthcare professional?

- Always, regardless of my family’s preference
- If my family want to inform me but has difficulty doing so, so they seek help from a healthcare professional
- If my family does not want to inform me
- Other (specify) ………………………………………………………………….

**19**. Which healthcare service or professional would you prefer to be informed by? (multiple answers possible)

- The genetics service that counseled/tested my family members (medical geneticist)
- The genetics service that counseled/tested my family members (genetic nurse)
- The genetics service closest to where I live (medical geneticist)
- The genetics service closest to where I live (genetic nurse)
- My family doctor
- My family nurse
- The public health-prevention service
- No particular preference
- Other (specify) ……………………………………………………………….

**20** How would you prefer to receive the information from a healthcare professional?

- In person
- By phone call
- By letter
- By email
- By text message (eg. WhatsApp)
- Other (specify)…………………………………………………………………

**21** I would prefer to be informed by a family member with whom I have no contact rather than not being informed at all

| Strongly agree | Agree | Neither agree nor disagree | Disagree | Strongly disagree |
| --- | --- | --- | --- | --- |

**22** I would prefer to be informed by a doctor/healthcare professional rather than not being informed at all.

| Strongly agree | Agree | Neither agree nor disagree | Disagree | Strongly disagree |
| --- | --- | --- | --- | --- |

**23** I would prefer to be informed by a doctor/healthcare professional rather than by a family member with whom I have no contact.

| Strongly agree | Agree | Neither agree nor disagree | Disagree | Strongly disagree |
| --- | --- | --- | --- | --- |

**24** I would prefer that the first communication comes by a family member with whom I have a close relationship.

| Strongly agree | Agree | Neither agree nor disagree | Disagree | Strongly disagree |
| --- | --- | --- | --- | --- |

**25** If you want to add any comments or share any information that you consider relevant, please use the space below:

………………………………………………………………………………………………………………………………………………..……

**Section 6: Disclosure of Personal Diagnosis**

Thank you for answering the questions about who you would prefer to be informed by. Now imagine that you are the first person in your family to have the genetic test. The following are questions about **how you would or would not want to share tha**t **information with your family.**

*There are no right or wrong answers. Select the option that best describes your point of view.*

**26**. If I were the first person in my family to be diagnosed with a hereditary disease, I would want the following family members to be informed:

- Only close family members (parents, siblings, children) (*Get to next item)*
- Only family members with whom I have regular contact *(Get to next item)*
- All my family members, even those I don’t have regular contact with (*Get to next item)*
- None of my family members *(Get to item 30)*

**27** If I was the first person in my family be diagnosed with a hereditary disease, I would personally inform my family members

| Strongly agree | Agree | Neither agree nor disagree | Disagree | Strongly disagree |
| --- | --- | --- | --- | --- |

**28** If I were the first person in my family to be diagnosed with a hereditary disease, I would want healthcare professionals to inform my family members

| Strongly agree | Agree | Neither agree nor disagree | Disagree | Strongly disagree |
| --- | --- | --- | --- | --- |

**29** If I were the first person in my family to be diagnosed with a hereditary disease, I would inform my family members with the help of healthcare professionals

| Strongly agree | Agree | Neither agree nor disagree | Disagree | Strongly disagree |
| --- | --- | --- | --- | --- |

**30** Why would you not want your family members to be informed?

………………………………………………………………………………………………………………………………………………..……

**31** If you would like to add any comments or share any information you find relevant, please use the space below

………………………………………………………………………………………………………………………………………………..……

**Section 7: Family Relationships**

Thank you for answering the queries regarding who would you rather inform in your family.

Now we would like you to tell us something about how you see your family in this moment. So we ask you YOUR personal point of view about your biological family *(those* relatives that are genetically related to you like parents, siblings and children)

For each question answer matching the proposition that better describes your family, from 1 (Very well) to 5 (Not at all)

Do not dwell too much on each question, but answers to all of them anyway.

**32.**

|  | Very well | Well | Partly | Not well | Not at all |
| --- | --- | --- | --- | --- | --- |
| In my family we talk to each other about things which matter to us |  |  |  |  |  |
| People often don’t tell each other the truth in my family |  |  |  |  |  |
| Each of us gets listened to in our family |  |  |  |  |  |
| It feels risky to disagree in our family |  |  |  |  |  |
| We find it hard to deal with everyday problems |  |  |  |  |  |
| We trust each other |  |  |  |  |  |
| It feels miserable in our family |  |  |  |  |  |
| When people in my family get angry they ignore each other on purpose |  |  |  |  |  |
| We seem to go from one crisis to another in my family |  |  |  |  |  |
| When one of us is upset they get looked after within the family |  |  |  |  |  |
| Things always seem to go wrong for my family |  |  |  |  |  |
| People in the family are nasty to each other |  |  |  |  |  |
| People in my family interfere too much in each other’s lives |  |  |  |  |  |
| In my family we blame each other when things go wrong |  |  |  |  |  |
| We are good at finding new ways to deal with things that are difficult |  |  |  |  |  |

**33** If you would like to add any comments or share any information you find relevant, please use the space below

………………………………………………………………………………………………………………………………………………..……

**Section 8: Socio-Demographic Characteristics**

We ask you finally to answer to some queries regarding socio-demographic aspects

**How old are you?**

_____ (number)

**Gender**

- Male
- Female
- I prefer to not declare it

**What is your current marital status?**

- Single
- Married
- Cohabiting
- Divorced

**Do you have children?**

- Yes
- No

**Do you plan to have children or to increase the family with others?**

- Yes
- No
- Maybe
- I do not know

**Which is your highest level of education?**

- Primary School Diploma
- Middle School Diploma
- High School Diploma
- Bachelor’s Degree
- Postgraduate Specialization
- Doctorate (PhD)

**What is your current employment status?**

- Paid employment
- Voluntary work
- Student
- Household responsibilities
- Retired
- Other (specify) ………………………………….


   **If you have a paid employment, do you work as a healthcare professional?**

- Yes
- No

  If “Yes” to the previous question

**Which one is your profession?**

- Healthcare assistant
- Biologist
- Chemist
- Dietitian
- Professional educator
- Pharmacist
- Physician
- Physiotherapist
- Dental hygienist
- Nurse
- Pediatric Nurse
- Speech therapist
- Medical doctor
- Odontologist
- Orthotic and ophthalmologic assistant
- Midwife
- Podiatrist
- Psychologist
- Audiometric technician
- Acoustic-aid technician
- Cardiovascular perfusionist
- Environment and Workplace Prevention Technician
- Neurophysiopathology technician
- Orthopaedic technician
- Technical of the psychiatric rehabilitation
- Biomedical laboratory technician
- X-ray technician
- Neuro and Psychomotor Therapist of Developmental Age
- Occupational Therapist
- Veterinarian

**How do you approach to religion?**

- Practicing Believer *(which religion? Next question)*
- Non-Practicing Believer *(which religion? Next question)*
- Non Believer *(go to question regarding “Residence area”)*

Other (specify): ____________________


**How would you define yourself:**

- Catholic christian
- Muslim
- Other (specify): ____________________


   **What is your current area of residence?**
   □ Abruzzo
   □ Basilicata
   □ Calabria
   □ Campania
   □ Emilia-Romagna
   □ Friuli-Venezia Giulia
   □ Latium
   □ Liguria
   □ Lombardy

   □ Marche
   □ Molise
   □ Piedmont
   □ Apulia
   □ Sardinia
   □ Sicily
   □ Tuscany
   □ Trentino South Tyrol
   □ Umbria
   □ Aosta Valley
   □ Veneto
   □ Residency outside Italy


  *Thank you for responding to our questionnaire!*

**Part 2**

**Table S1. The four genetic literacy (awareness) -based questions**

|  | Nurse participants  (n=315) | | | | Non-nurse participants  (n=185) | | | |
| --- | --- | --- | --- | --- | --- | --- | --- | --- |
| **Genetic literacy (awareness)** | No | | Yes | | No | | Yes | |
|  | n | % | n | % | n | % | n | % |
| *Determining the risk or probability of developing a specific disease* | 77 | 17.0 | 238 | 29.5 | 27 | 15.6 | 158 | 27.9 |
| *Determining how a disease should be managed following diagnosis* | 151 | 33.4 | 164 | 20.3 | 67 | 38.7 | 118 | 20.8 |
| *Determining which medications may or may not be effective for an individual* | 144 | 31.9 | 171 | 21.2 | 55 | 31.8 | 130 | 22.9 |
| *Determining the probability of transmitting a hereditary disease to offspring* | 80 | 17.7 | 235 | 29.1 | 24 | 13.9 | 161 | 28.4 |

**Table S2. Comparisons of groups with different "Wanting to Know" responses among non-nurse HCPs**

|  |  | **Wanting to be informed about at least one condition versus none** | | | |  | **Wanting to be informed about three conditions versus one or two conditions** | | | |  |
| --- | --- | --- | --- | --- | --- | --- | --- | --- | --- | --- | --- |
|  |  | None | | At least one | |  | 1 or 2 conditions | | All 3 conditions | |  |
|  |  | (n=9) | | (n=177) | |  | (n=48) | | (n=129) | |  |
| **Characteristics of the study participants** | |  |  |  |  |  |  |  |  |  |  |
|  |  | Mean | SD | Mean | SD | p-value | Mean | SD | Mean | SD | p-value |
| **Age** |  | 43.1 | 13.3 | 42.7 | 10.9 | 0.904 | 42.9 | 10.8 | 42.6 | 11.0 | 0.847 |
|  |  | n | % | n | % |  | n | % | n | % |  |
| **Gender*** | |  |  |  |  |  |  |  |  |  |  |
|  | *Male* | 2 | 25.0 | 26 | 14.7 | 0.348 | 6 | 12.5 | 20 | 15.5 | 0.812 |
|  | *Female* | 6 | 75.0 | 151 | 85.3 |  | 42 | 87.5 | 109 | 84.5 |  |
| **Marital Status** | |  |  |  |  |  |  |  |  |  |  |
|  | *Single* | 2 | 22.2 | 41 | 23.2 | 0.972 | 10 | 20.8 | 31 | 24.0 | 0.374 |
|  | *Married* | 5 | 55.6 | 84 | 47.5 |  | 21 | 43.8 | 63 | 48.8 |  |
|  | *Cohabiting* | 2 | 22.2 | 45 | 25.4 |  | 15 | 31.3 | 30 | 23.3 |  |
|  | *Divorced* | 0 | 0.0 | 6 | 3.4 |  | 1 | 2.1 | 5 | 3.9 |  |
|  | *Widowed* | 0 | 0.0 | 1 | 0.6 |  | 1 | 2.1 | 0 | 0.0 |  |
| **Children** | |  |  |  |  |  |  |  |  |  |  |
|  | *No* | 3 | 33.3 | 82 | 46.3 | 0.512 | 22 | 45.8 | 60 | 46.5 | 1.000 |
|  | *Yes* | 6 | 66.7 | 95 | 53.7 |  | 26 | 54.2 | 69 | 53.5 |  |
| **Highest Level of Education** | |  |  |  |  |  |  |  |  |  |  |
|  | *Middle School Diploma* | 0 | 0.0 | 2 | 1.1 | 0.126 | 0 | 0.0 | 2 | 1.6 | 0.997 |
|  | *High School Diploma* | 2 | 22.2 | 16 | 9.0 |  | 6 | 12.5 | 10 | 7.8 |  |
|  | *Bachelor's Degree* | 5 | 55.6 | 76 | 42.9 |  | 20 | 41.7 | 56 | 43.4 |  |
|  | *Postgraduate Specialization* | 2 | 22.2 | 76 | 42.9 |  | 19 | 39.6 | 57 | 44.2 |  |
|  | *Doctorate (PhD)* | 0 | 0.0 | 7 | 4.0 |  | 3 | 6.3 | 4 | 3.1 |  |
| **Religious Orientation** | |  |  |  |  |  |  |  |  |  |  |
|  | *Believer (Practing/Non-practing)* | 5 | 55.6 | 112 | 63.3 | 0.728 | 35 | 72.9 | 77 | 59.7 | 0.117 |
|  | *Non-Believer/Agnostic* | 4 | 44.4 | 65 | 36.7 |  | 13 | 27.1 | 52 | 40.3 |  |
| **Current Area of Residence** | |  |  |  |  |  |  |  |  |  |  |
|  | *Northern Italy* | 8 | 88.9 | 155 | 87.6 | 0.791 | 43 | 89.6 | 112 | 86.8 | 0.241 |
|  | *Central Italy* | 1 | 11.1 | 10 | 5.6 |  | 1 | 2.1 | 9 | 7.0 |  |
|  | *Souther Italy and Islands* | 0 | 0.0 | 11 | 6.2 |  | 3 | 6.3 | 8 | 6.2 |  |
|  | *Abroad* | 0 | 0.0 | 1 | 0.6 |  | 1 | 2.1 | 0 | 0.0 |  |
|  |  | Mean | SD | Mean | SD |  | Mean | SD | Mean | SD |  |
| **Genetic literacy (awareness)** | | 3.1 | 1.3 | 3.0 | 1.4 | 0.892 | 3.0 | 1.5 | 3.1 | 1.4 | 0.890 |
|  |  |  |  |  |  |  |  |  |  |  |  |
| **Family relationship (Score-15)** | | 2.0 | 0.7 | 2.0 | 0.6 | 0,996 | 2.0 | 0.6 | 2.0 | 0.6 | 0.675 |
| *Legend*  **One respondent who selected 'prefer not to disclose' for sex were excluded and treated as missing data* | | | | | | | | | | | |

**Table S3. Comparison between willingness to know about genetic risk and willingness to undergo genetic testing**

|  | | **Willingness to undergo genetic testing** | | | |
| --- | --- | --- | --- | --- | --- |
| **Willingness to know** | | ***Nurse participants*** | | ***Non-nurse participants*** | |
|  |  | ***No***  ***n (%)*** | ***Yes***  ***n (%)*** | ***No***  ***n (%)*** | ***Yes***  ***n (%)*** |
|  |  | Cystic Fibrosis | | Cystic Fibrosis | |
| Cystic Fibrosis | No | 27 (81.8) | 6 (18.2) | 20 (90.9) | 2 (9.1) |
|  | Yes | 7 (2.6) | 259 (97.4) | 9 (5.8) | 146 (94.2) |
|  |  | Hereditary Cancer | | Hereditary Cancer | |
| Hereditary Cancer | No | 6 (100.0) | 0 (0.0) | 2 (100.0) | 0 (0.0) |
|  | Yes | 4 (1.4) | 289 (98.6) | 2 (1.1) | 173 (98.9) |
|  |  | Early-Onset Alzheimer's disease | | Early-Onset Alzheimer's disease | |
| Early-Onset Alzheimer's disease | No | 38 (82.6) | 8 (17.4) | 32 (94.1) | 2 (5.9) |
|  | Yes | 9 (3.6) | 244 (96.4) | 6 (4.2) | 137 (95.8) |

**Table S4. Preferences for the source, situation, and method of receiving genetic risk information (non-nurse participants)**

| **Items** | **n** | **%** |
| --- | --- | --- |
| **Item 13. In your opinion, who has the moral responsibility to inform you?** | | |
| *All (self, relatives, healthcare professionals)* | 28 | 15.8 |
| *Only self* | 2 | 1.1 |
| *Only relatives* | 16 | 9.0 |
| *Only healthcare professionals* | 19 | 27.9 |
| *Both relatives and healthcare professionals* | 89 | 43.2 |
| *Both self and relatives* | 9 | 5.1 |
| *Both self and healthcare professionals* | 11 | 6.2 |
| *No one/Not known* | 3 | 1.7 |
| **Item 15 – Preferred family member to receive the information from*:** | | |
| A family member who has already taken the genetic test | 61 | 48.4 |
| A family member with whom I have a close relationship | 14 | 11.1 |
| A family member I am in regular contact with | 2 | 1.6 |
| No particular preference | 49 | 38.9 |
| **Item 18 – Preferred situation to be contacted and informed by a healthcare professional**:** | | |
| Always, regardless of my family's preference | 103 | 69.1 |
| If my family wants to inform me but has difficulty doing so, so they seek help from a healthcare professional | 45 | 30.2 |
| If my family does not want to inform me | 1 | 0.7 |
| **Item 16 – Preferred method of receiving information from a family member***:** | | |
| Message (e.g. WhatsApp) | 2 | 1.6 |
| Email | 0 | 0.0 |
| Letter | 0 | 0.0 |
| Phone call | 10 | 8.1 |
| Video call | 1 | 0.8 |
| In person | 111 | 89.5 |
| **Item 20 – Preferred method of receiving information from a healthcare professional****:** | | |
| Message (e.g. WhatsApp) | 0 | 0.0 |
| Email | 4 | 2.8 |
| Letter | 0 | 0.0 |
| Phone call | 14 | 10.1 |
| Video call | 0 | 0.0 |
| In person | 121 | 87.1 |

**Table S5 Preferences for communication (non-nurse participants)**

|  |  | **Strongly disagree** | **Disagree** | **Neither agree nor disagree** | **Agree** | **Strongly agree** |
| --- | --- | --- | --- | --- | --- | --- |
| **Items** |  | **n(%)** | **n(%)** | **n(%)** | **n(%)** | **n(%)** |
| **(a) Preferences for communication from the perspective of a person who could receive the information** | | | | | | |
| Item 14 | I would prefer to be informed by a family member | 6 (3.4) | 8 (4.5) | 37 (20.9) | 64 (36.2) | 62 (35.0) |
| Item 17 | I would prefer to be contacted and informed by a healthcare professional | 0 (0.0) | 3 (1.7) | 25 (14.1) | 63 (35.6) | 86 (48.6) |
| Item 21 | I would prefer to be informed by a family member with whom I have no contact rather than not being informed at all | 8 (4.5) | 10 (5.6) | 12 (6.8) | 50 (28.2) | 97 (54.8) |
| Item 22 | I would prefer to be informed by a doctor/healthcare professional rather than not being informed at all | 0 (0.0) | 0 (0.0) | 5 (2.8) | 40 (22.6) | 132(74.6) |
| Item 23 | I would prefer to be informed by a doctor/healthcare professional rather than by a family member with whom I have no contact | 5 (2.8) | 6 (3.4) | 42 (23.7) | 48 (27.1) | 76 (42.9) |
| Item 24 | I would prefer to be informed first by a family member with whom I have a close relationship | 7 (4.0) | 15 (8.5) | 69 (39.0) | 59 (33.3) | 27(15.3) |
| **(b) Preferences for communication from the perspective of the informant** | | | | | | |
| Item 27 | If I was the first person in my family be diagnosed with a hereditary disease, I would personally inform my family members | 0 (0.0) | 1 (0.6) | 12 (6.8) | 52 (29.4) | 112 (63.3) |
| Item 28 | If I were the first person in my family to be diagnosed with a hereditary disease, I would want healthcare professionals to inform my family members | 2 (1.1) | 23 (13.0) | 61 (34.5) | 54 (30.5) | 37 (20.9) |
| Item 29 | If I were the first person in my family to be diagnosed with a hereditary disease, I would inform my family members with the help of health professionals | 1 (0.6) | 3 (1.7) | 22 (12.4) | 64 (36.2) | 87 (49.2) |

**Table S6 Item19 - Preferred healthcare source for receiving genetic information (multiple choices allowed)**

|  | **Nurse participants**  (N=299) | | | | **Non-nurse participants**  (N=177) | | | |
| --- | --- | --- | --- | --- | --- | --- | --- | --- |
|  | Yes | | No | | Yes | | No | |
|  | n | % | n | % | n | % | n | % |
| The genetics service that counseled/tested my family members (medical geneticist) | 168 | 56.2 | 131 | 43.8 | 109 | 61.6 | 68 | 38.4 |
| The genetics service that counseled/tested my family members (genetic nurse) | 115 | 38.5 | 184 | 61.5 | 18 | 10.2 | 159 | 89.8 |
| The genetics service closest to where I live (medical geneticist) | 60 | 20.1 | 239 | 79.9 | 30 | 16.9 | 147 | 83.1 |
| The genetics service closest to where I live (genetic nurse) | 49 | 16.4 | 250 | 83.6 | 10 | 5.6 | 167 | 94.4 |
| My family doctor | 81 | 27.1 | 218 | 72.9 | 51 | 28.8 | 126 | 71.2 |
| My family nurse | 36 | 12.0 | 263 | 88.0 | 5 | 2.8 | 172 | 97.2 |
| The public health-prevention service | 48 | 16.1 | 251 | 83.9 | 36 | 20.3 | 141 | 79.7 |
| No particular preference | 60 | 20.1 | 239 | 79.9 | 30 | 16.9 | 147 | 83.1 |

**Table S7 Ranking of respondents’ preferences for receiving genetic risk information: family members versus healthcare professionals**

| ***Participants preferred to receive genetic risk information from…*** | **Nurse participants**  (N=299) | | **Non-nurse participants**  (N=177) | |
| --- | --- | --- | --- | --- |
|  | **n** | **%** | **n** | **%** |
| Family members | 122 | 40.8 | 72 | 40.7 |
| Healthcare professionals | 46 | 15.4 | 37 | 20.9 |
| Equally from both (including don’t know) | 12 | 4.0 | 1 | 0.5 |
| Disagree with both | 119 | 39.8 | 67 | 37.9 |

**Table S8 Item 26 - Participants preferred which family members should be informed about genetic risk**

|  | **Nurse participants**  (N=299) | | **Non-nurse participants**  (N=177) | |
| --- | --- | --- | --- | --- |
|  | **n** | **%** | **n** | **%** |
| Only first-degree relatives (parents, siblings, children) | 88 | 29.4 | 54 | 30.5 |
| Only relatives with whom I have regular contact | 36 | 12.0 | 13 | 7.3 |
| All family members, including those with whom I have no regular contact | 171 | 57.2 | 110 | 62.1 |
| None of my family members | 4 | 1.3 | 0 | 0.0 |

**Table S9 Ranking of respondents’ preferences for informing their relatives about own genetic risk**

| ***If I were the first person in my family to be diagnosed with a hereditary disease…*** | **Nurse participants**  **(N=295)*** | | **No**n-n**urse participants**  **(N=177)** | |
| --- | --- | --- | --- | --- |
|  | **n** | **%** | **n** | **%** |
| I would personally inform my family members | 187 | 63.4 | 104 | 58.8 |
| I would want healthcare professionals to inform relatives | 29 | 9.8 | 15 | 8.5 |
| Equally from family members or healthcare professionals both (including don’t know) | 77 | 26.1 | 58 | 32.7 |
| Disagree with both | 2 | 0.7 | 0 | 0.0 |

*4 missing
